# ﻿*Silenevanchingshanensis* (Caryophyllaceae) a new species from Southwest China

**DOI:** 10.3897/phytokeys.189.79631

**Published:** 2022-02-15

**Authors:** Feng Yang, Jin-Li Liu, Ping-Ping Li, Huan-Chong Wang

**Affiliations:** 1 School of Life Sciences, Yunnan University, Kunming 650500, Yunnan, China; 2 School of Ecology and Environmental Science, Yunnan University, Kunming 650500, Yunnan, China; 3 Herbarium of Yunnan University, Kunming 650091, Yunnan, China

**Keywords:** Endemism, Guizhou, *Silene*, *Silenemorrisonmontana*, *Silenehupehensis*

## Abstract

*Silenevanchingshanensis* (Caryophyllaceae), a new species from Fanjingshan Mountain in Guizhou (southwest China) is described and illustrated. It is morphologically similar to *S.morrisonmontana* and *S.hupehensis*, from which it can be easily distinguished by having pubescent stems usually 10–15 cm long, linear-oblanceolate leaves 3–6 cm × 3–6 mm, often 2–5-flowered cymes, pink or violet petals and narrowly ovoid capsules.

## ﻿Introduction

*Silene* L. (*Sileneae* DC., Caryophylloideae Arnott, Caryophyllaceae Juss.) is the largest genus of the carnation family, comprising 700 to 870 species (Mabberley 2017; Jafari et al. 2020), mostly occurring in temperate regions and subtropical mountains of the Northern Hemisphere (Zhou et al. 2001; Oxelman et al. 2011). The centre of its species diversity is observed in Western Asia and the Mediterranean area, but areas of Central Asia are also highly diverse (see, for example, Jafari et al. 2020). Taxonomically, *Silene* represents a notoriously difficult genus, having a high species-richness, widespread distribution, broad morphological variations and the complex genetic background. Its generic delimitation has been controversial (Oxelman and Lidén 1995; Jafari et al. 2020) with some authors lumping many members into the genus (e.g. Greuter 1995; Desfeux and Lejeune 1996; Jafari et al. 2020), whereas others support separation of *Agrostemma*, *Atocion*, *Eudianthe*, *Heliosperma*, *Petrocoptis* and *Viscaria* (e.g. Oxelman and Lidén 1995; Oxelman et al. 1997, 2001; Popp and Oxelman 2004; Frajman et al. 2009a, b; Greenberg and Donoghue 2011). In addition to taxonomic research, the genus *Silene* is also difficult from the nomenclatural point as highlighted, for example, by Iamonico (2018, 2021).

Concerning China, the first comprehensive revision of the genus *Silene* was carried out by Tang (1996) who recognised 131 species (including two subspecies and 17 varieties). In the most recent treatment by Zhou et al. (2001), 110 species were accepted, of which 67 are endemic. *Silene* taxa can be found throughout the country, mostly being found in the north-western and south-western provinces, with more than 60 species in the Hengduan Mountains (Zhou 1983; Wu 1993; Zhuang 1995; Tang 1996; Wu et al. 2003).

As part of the taxonomic revision of *Silene* in the Sino-Himalayan Region for the *Flora of Pan-Himalayas*, an undescribed species was found and is proposed here.

## ﻿Materials and methods

The new species was studied both in the field and at herbaria. The collections housed at CDBI, KUN, PE, PYU, XTBG and YUKU (acronyms according to Thiers 2022), as well as digital images available at JSTOR Global Plants (http://plants.jstor.org/) and at the Chinese Virtual Herbarium (http://www.cvh.ac.cn/), were examined. Pertinent taxonomic literature (e.g. Xiao and Xie 1982; Zhuang 1995; Tang 1996; Zhou et al. 2001) were extensively consulted. Morphological studies were carried out on dried material under a stereomicroscope (Olympus SZX2, Tokyo, Japan) and measurements were made using a ruler and a metric vernier caliper.

## ﻿Taxonomy

### ﻿ Silene vanchingshanensis

Taxon classificationPlantaeCaryophyllalesCaryophyllaceae

C.Y.Wu ex Huan C. Wang & Feng Yang sp. nov.

5B48E877-DDBD-5368-A461-FA380F86A158

urn:lsid:ipni.org:names:77254897-1

Figs 1 , 2 , 3

#### Type.

China. Guizhou Province: Jiangkou County, summit of the Fanjingshan Mountain, 27°54'51"N, 108°41'35"E, steep cliffs or rock crevices, alt. 2,450–2,500 m, 10 July 2021, *Feng Yang & Jing-Li Liu JK 12775* (holotype YUKU02074621!; isotypes YUKU02074622!, YUKU02074623!, YUKU02074624!, YUKU02074625!, YUKU02074643!).

**Figure 1.**
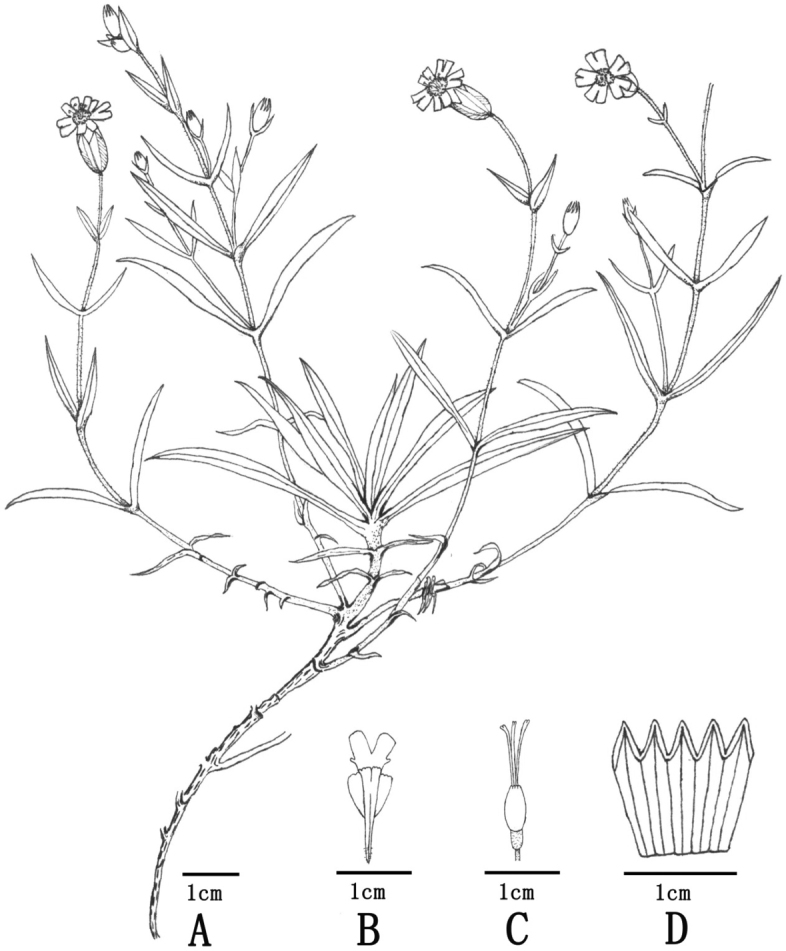
*Silenevanchingshanensis* (Drawn from the holotype by Jing-Li Liu) **A** habit **B** petal **C** pistil **D** calyx.

#### Diagnosis.

*Silenevanchingshanensis* is similar to *S.morrisonmontana*, from which it differs by its shape and size of leaves (linear-oblanceolate, 3–6 cm × 3–6 mm vs. linear, 2–7 cm × 2–3 mm), cymes (often 2–5-flowered vs. usually solitary) and colour of petals (pink or violet vs. white).

**Figure 2.**
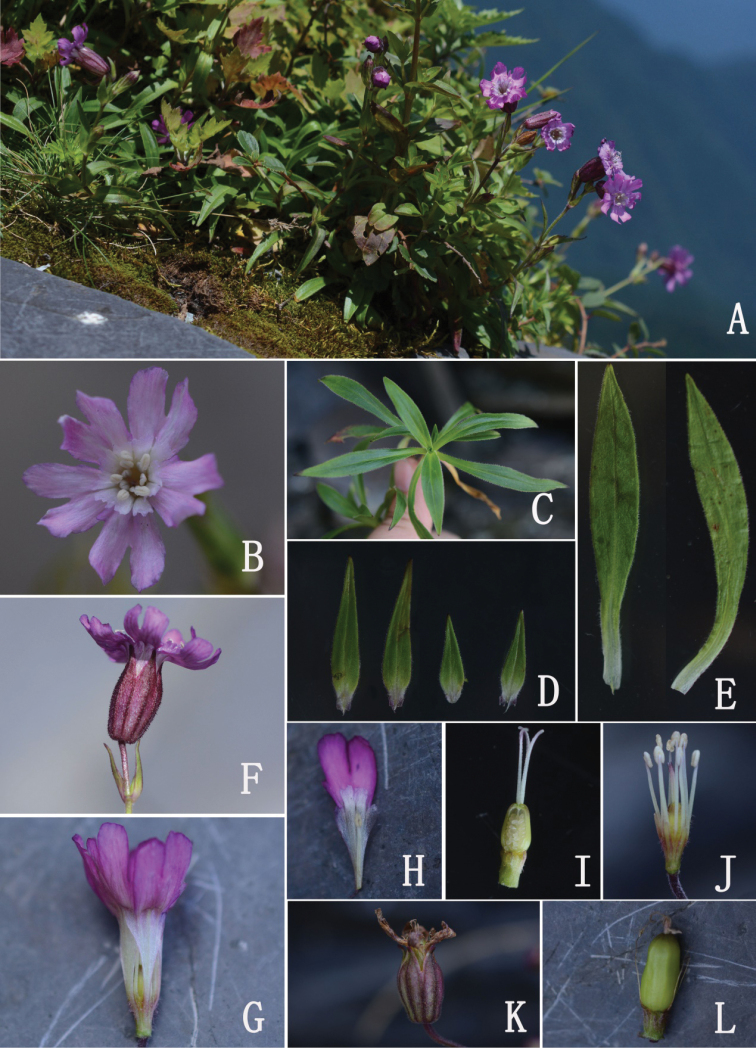
*Silenevanchingshanensis***A** habit **B** flower (front view) **C** basal leaves **D** cauline leaves and bracts **E** basal leaves **F** flower (side view, showing the calyx and pedicel) **G** dissected flower (showing the androgynophore and claws) **H** petal (showing the claw, auricles and coronal scales) **I** pistil and androgynophore **J** stamens, pistil and androgynophore **K** calyx after anthesis **L** immature capsule.

#### Description.

Herbs perennial. Rhizomes slender, creeping, branched. Stems caespitose, ascending, 10–15 cm long, slender, pubescent, usually with clustered sterile shoots at the base. Basal leaves linear-oblanceolate, 3–6 cm long, 3–6 mm wide, base cuneate, attenuate into petiole, connate, cylindrical, apex acuminate, margin ciliate, mid-vein prominent; cauline leaves usually 4–6 pairs, sessile, lanceolate to linear-oblanceolate, 2–4 cm long, 3–4 mm wide, apex acuminate, margin ciliate. Cymes often 2–5-flowered, flowers rarely solitary. Flowers slightly nodding; pedicel densely hairy, 8–25 mm long; bracts lanceolate, 10–15 mm long, ca. 2 mm wide. Calyx campanulate, ca. 12 mm long, 5–8 mm in diameter, base rounded, longitudinal veins violet, converging at apex, veins hairy; calyx teeth narrowly triangular, 3–4 mm long, margin ciliate, apex acute to acuminate. Androgynophore 2–3 mm long, pubescent. Petals pink or violet, 1.5–2.0 cm long; claws saccate-oblanceolate, ciliate at base; auricles orbicular, sometimes obscurely laciniate; limbs exserted beyond calyx, obovate, 6–9 mm long, bifid, rarely deeply lobed to middle; lobes narrowly elliptic or ovate, sometimes with one obtuse tooth on each lateral side; coronal scales flabellate, ca. 1 mm long, white with a little tint of violet, laciniate at apex. Stamens 10, slightly exserted, filaments hairy at base. Styles usually 3, sometimes 5, included. Capsule narrowly ovoid, 12–15 mm long, slightly equal to persistent sepals. Seeds reniform.

**Figure 3.**
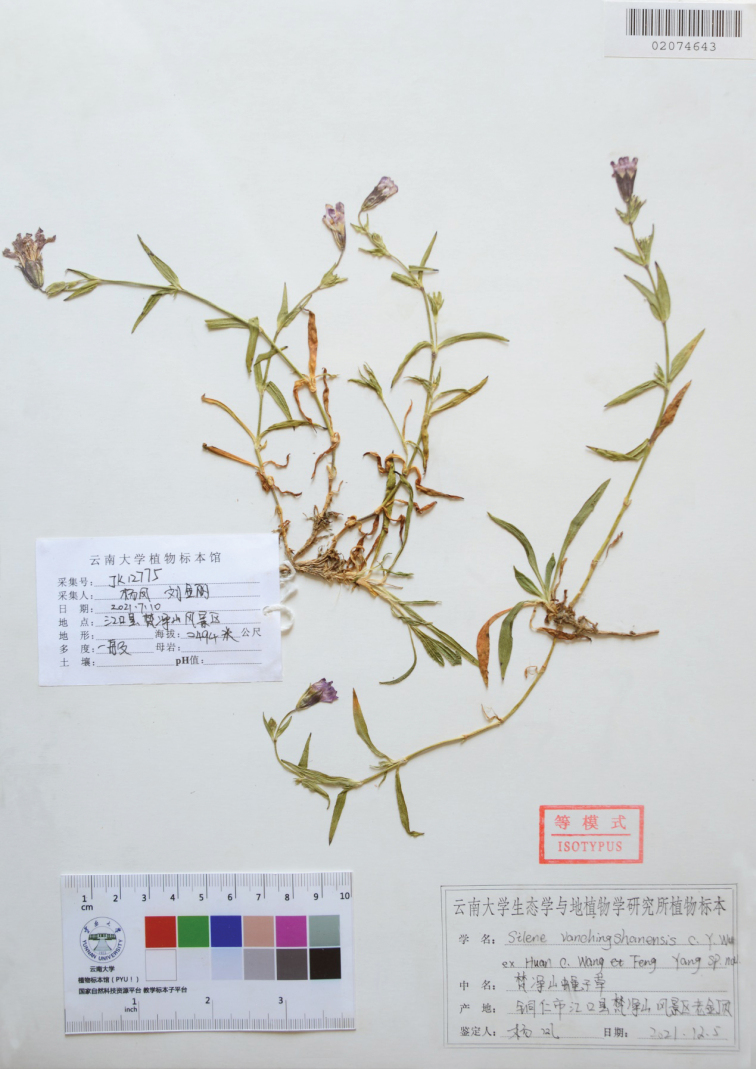
An isotype of *Silenevanchingshanensis* (YUKU 02074643).

#### Phenology.

Flowering and fruiting times from July to September.

#### Etymology.

The specific epithet *vanchingshanensis* is derived from the type locality “Vanchingshan”, a variant name for Fanjingshan Mountain.

#### Distribution and habitat.

*Silenevanchingshanensis* is currently known only from *locus classicus* (Fanjingshan Mountain), a famous scenic resort in Guizhou, southwest China. The species grows on cliffs or rock crevices of the mountain summit at elevations of 2,100–2,500 m.

#### Taxonomic notes.

The name *Silenevanchingshanensis* was first proposed by the Chinese botanist Zhengyi Wu in 1988 on the label of a KUN specimen (*C. P. Jian et al. 32080*). However, no name was formally published.

According to the taxonomic treatment of Chinese *Silene* by Tang (1994), *S.vanchingshanensis* should be assigned to Silenesect.Morrisonmontanae C. L. Tang (synonymised in Silenesect.Physolychnis (Benth.) Bocquet in Jafari et al. (2020)) due to its cymes 2–5-flowered and campanulate calyx. *S.vanchingshanensis* is mostly similar to *S.morrisonmontana* (Hayata) Ohwi & H.Ohashi, but differs from the latter by its leaves linear-oblanceolate (vs. linear), 3–6 cm × 3–6 mm (vs . 2–7 cm × 2–3 mm), cymes (1–) 2–5-flowered (vs. usually solitary) and petals pink or violet (vs. white). The distribution areas of these two species are separated: *S.vanchingshanensis* is endemic to Guizhou, while *S.morrisonmontana* is only found in Taiwan. *S.vanchingshanensis* is also similar to *S.hupehensis* C. L. Tang, but clearly differs from the latter by its stems with hairs, 10–15 cm (vs. glabrous, 10–30 cm) long, leaves linear-oblanceolate (vs. narrowly linear), 3–6 cm × 3–6 mm (vs. 5–8 cm × 2–3.5 mm) and capsule narrowly ovoid (vs. ovoid), 12–15 mm (vs. 6–8 mm) long. A detailed morphological comparison between these three species is summarised in Table 1.

**Table 1.** Morphological comparison of *S.vanchingshanensis*, *S.morrisonmontana* and *S.hupehensis*.

Characters	Species		
	*S.vanchingshanensis*	*S.morrisonmontana*	*S.hupehensis*
**Stems (cm)**	10–15	10–15	10–30
**Leaves (cm × mm)**	linear-oblanceolate, 3–6 × 3–6	narrowly linear, 2–7 × 2–3	narrowly linear, 5–8 × 2–3.5
**Inflorescence**	often 2–5-flowered cymes, rarely solitary	flowers solitary	often 2–5-flowered cymes, rarely solitary
**Calyx (cm × mm)**	campanulate, 1.2 × 5–8, veins hairy	cylindrical-campanulate, swollen, 1.4–1.8 × 10–12, veins hirsute	campanulate, 1.2–1.5 × 3.5–7, veins glabrous
**Petals**	pink or violet, limbs obovate, 6–9 mm long, bifid, lobes narrowly elliptic or ovate	white, limbs obovate, 4–6 mm long, shallowly bifid; lobes narrowly elliptic or ovate	pink, limbs obovate or broadly ovate, 7–9 mm long, shallowly bifid, lobes nearly orbicular
**Fruit**	narrowly ovoid, 12–15 mm	narrowly ovoid, 8–10 mm	ovoid, 6–8 mm
**Distribution**	Guizhou, southwest China	Taiwan, east China	Gansu, Henan, Hubei, Shaanxi, Sichuan, central to southwest China

#### Additional specimens examined.

*Silenevanchingshanensis* (paratypes). **China. Guizhou**: Jiangkou County, Fanjingshan Mountain, 15 August 2003, *S. Z. He et al. 0308038* (GZTM), ibid., Jingding, alt. 2,150 m, 25 September 1963, *C. P. Jian et al. 32080* (KUN), ibid., collection time unknown, *s. n. 51495* (IBSC).

*Silenemorrisonmontana*. **China. Taiwan**: Hsinchu City, Wufeng village, Sheipa National Park, Tapachienshan, 24°27'47"N, 121°15'29"E, on shady rocky slope, alt. ca. 3,400 m, 7 September1993, *C. L. Huang et al. 103* (HAST), Taichung City, Wuling, on route from 369 Lodge to Hsuehshan Peak, alt. ca. 3,884 m, 2 August 1991, *D. S. HSU & Moore*, *S. J. 723* (HAST), Nantou County, Jenai village, Chilailishan, alt. 3,330 m, 4 September 1998, *T. Y. A. Yang No. 11253* (PE).

*Silenehupehensis*. **China. Henan**: Luanchuan County, Laojunshan, 24 July 2006, *Chang-Shan Zhu 2006100* (HITBC). **Hubei**: Shengnongjia Forest District, along the road between Guanmenshan and Xiaoshennongjia, 31°30'N, 110°30'E, 10 September 1980, *1980 Sino-Amer. Exped. No 973* (PE). **Shaanxi**: Mei County, Taibai Mountain, Fangyang temple, hillsides and meadows, alt. 3,000 m, 9 August 1977, *You-Hao Guo & Zhi-Xing Hu 489* (IBSC). **Sichuan**: Shimian County, on the way from Jiziping to Xishan, 29°04'29"N, 102°11'22"E, alt. 2,961 m, 31 July 2007, *Ji-Pei Yue Yue-07160* (KUN).

## Supplementary Material

XML Treatment for Silene vanchingshanensis
